# Effects of Lateralization of Language on Cognition Among Left-Handers

**DOI:** 10.1162/nol_a_00165

**Published:** 2025-04-24

**Authors:** Cristina Cano-Melle, Esteban Villar-Rodríguez, María Baena-Pérez, María Antonia Parcet, César Avila

**Affiliations:** Neuropsychology and Functional Neuroimaging, Universitat Jaume I, Castellón de la Plana, Spain

**Keywords:** cognitive skills, hemispheric language dominance, left-handedness, phenotypes of atypical language

## Abstract

Atypical language lateralization is associated with a different organization of the entire brain. However, it remains unknown whether this cerebral organization is linked to differences in cognitive task performance. In this study, several neuropsychological tests requiring fast processing speed were administered to left-handed participants, classified based on their language lateralization: left language dominance (*n* = 48), bilateral (*n* = 15), and right language dominance (*n* = 23). A factor analysis was conducted to derive three cognitive function dimensions: reading, articulation, and verbal reasoning; spatial processing; and interference/inhibition. The results showed that right language dominance was associated with poorer overall performance, particularly on tasks related to spatial processing, reading, articulation, and verbal reasoning. We conclude that the atypical development of language lateralization is accompanied by lower cognitive skills in tasks requiring speed of processing and interhemispheric connectivity.

## INTRODUCTION

Advances in neuroscience have provided key insights into understanding how the brain functions. One of the most relevant aspects of brain organization is the lateralization of various cognitive functions, which may predominantly occur in the left hemisphere, the right hemisphere, or both (Ocklenburg & Güntürkün, 2024). Among these functions, the lateralization of language production and comprehension in the left hemisphere is the most extensively studied in both clinical and basic research (Price, 2012). Various studies using neuroimaging techniques have revealed that 94%–96% of the right-handed individuals and 76%–78% of the left-handed individuals exhibit left lateralization of language function (Mazoyer et al., 2014; Pujol et al., 1999; Springer et al., 1999; Szaflarski et al., 2002). Not only that, but typically left-lateralized left-handers, when compared to typically left-lateralized right-handers, present reduced functional lateralization for diverse cognitive functions including language and non-language functions (Johnstone et al., 2021).

There is ongoing debate regarding how atypical lateralization of language and reduced lateralization affect the distribution and cognitive processing of other functions. Over the years, two contrasting hypotheses have been proposed to address this issue (see Badzakova-Trajkov et al., 2016, for a review). On the one hand, the Causal hypothesis suggests a mirror organization in the lateralization of functions, following a snowball mechanism (Hellige, 1990; Kosslyn, 1987). According to this model, for cognitive functions typically lateralized to different hemispheres—such as language (left lateralization) and visuospatial attention (right lateralization)—atypical lateralization of one function (e.g., language lateralized to the right hemisphere) would lead to the atypical lateralization of the other (e.g., visuospatial attention lateralized to the left hemisphere). On the other hand, the Statistical hypothesis posits that each cognitive function lateralizes independently, framing atypical language lateralization as a statistical phenomenon (Bryden et al., 1983). This model suggests that two cognitive functions typically lateralized to different hemispheres may crowd into the same hemisphere, potentially leading to neurobehavioral costs due to competition for shared processing resources (Gerrits et al., 2020).

Evidence supporting these theories is mixed. Most studies employing functional magnetic resonance imaging (fMRI) in left-handers have generally yielded results consistent with the Causal hypothesis at the group level (Cai et al., 2013; Villar-Rodríguez, Cano-Melle, et al., 2024; Villar-Rodríguez, Davydova, et al., 2024; Zago et al., 2016). However, these studies have also identified a substantial proportion of individual cases in which two functions, typically associated with different hemispheres, coexist within the same hemisphere. Additionally, research that includes both right- and left-handers has reported findings more consistent with the Statistical hypothesis (see Badzakova-Trajkov et al., 2016, for a review), as the correlation between the lateralization of two distinct functions remains unclear. Consequently, neither the Causal nor the Statistical hypothesis alone can fully account for the observed data, and, recently proposed frameworks have introduced the idea that each brain function lateralizes according to independent probabilistic biases. These frameworks suggest that certain mechanisms or events—possibly of genetic origin—may modulate these biases, allowing for variation or even reversal of lateralization patterns among genotypically distinct subpopulations (Gerrits, 2024; McManus, 2022).

In fact, there is evidence that exceptional brain conditions that facilitate crowding such as a weak lateralization of functions (Mellet et al., 2014) or an abnormal segregation of functions in the brain (Gerrits et al., 2020) are associated with lower cognitive performance. Given the significant role of language function in brain organization, studies have explored the impact of language lateralization on overall brain activity, behavioral task performance, and even neurological disorders. Several neurodevelopmental and psychiatric disorders, including schizotypy, autism, dyslexia, and epilepsy, may be more prevalent in individuals with atypical language lateralization compared to the general population (Eyler et al., 2012; Jouravlev et al., 2020; Ocklenburg & Güntürkün, 2024; Sommer et al., 2001; Villar-Rodríguez, Cano-Melle, et al., 2024). These findings raise the question of whether an atypical lateralization of language might be associated with differences in cognitive performance among healthy individuals. In this regard, the different research conducted in healthy individuals did not reveal clear differences when reporting cognitive performance as a function of lateralization of language (see Quin-Conroy et al., 2024, for a recent review). A study by Knecht et al. (2001) found no significant differences between typical and atypical groups across various measures, including self-reports of language use, academic achievement, artistic talents, and cognitive tests assessing verbal fluency, intelligence, and picture-word verification. However, research conducted with children has shown that those with atypical lateralization of language experience greater difficulties in vocabulary and nonword reading tasks (Groen et al., 2012), and exhibit lower verbal IQ scores (Everts et al., 2009; Lidzba et al., 2011). More recent studies have not found performance differences in individuals with atypical language lateralization, but have identified differences in specific subgroups of said individuals. In a large sample of right- and left-handed individuals with typical and atypical language lateralization, Mellet et al. (2014) explored 12 tests involving language, verbal memory, and spatial cognition. The results indicated that individuals with weak language lateralization (but not those with strong atypical lateralization) performed poorly across all three cognitive components. The authors suggested that individuals with atypical patterns of language organization may be associated with complex neural distribution and poor interhemispheric connections. Taking a different approach, Gerrits et al. (2020) studied the brain organization of different cognitive functions in left-handed participants with left and right dominance for language. The organization of functions was mostly consistent with the causal theory for all individuals. This study did not find cognitive differences in any domain between individuals with typical and atypical dominance of language, but they found the left-handed participants (*n* = 10) who deviated by two or more functions from this pattern exhibited lower cognitive performance. The authors hypothesized that the atypical coexistence of functions in the same hemisphere (crowding effect) could explain the differences in performance.

Therefore, results in adult healthy participants are scarce and inconclusive in demonstrating limitations in cognitive performance associated with atypical lateralization of language, especially beyond language functions. A recent review by Quin-Conroy et al. (2024) suggested two possible factors accounting for this lack of clarity. First, sample size in most of the studies is small, compromising their statistical power and, in turn, the solidity of results. The second relevant point is the lack of differentiation between atypical phenotypes, not isolating the role of right lateralization of language (entailing a mirrored organization) and bilateral organization of language (which entails interhemispheric transfer during cognitive tasks).

Also relevant is the lack of a hypothesis about the cerebral mechanisms underlying potential cognitive processing differences. Recent evidence has suggested that a greater volume in the corpus callosum might play a role as a main determinant of atypical lateralization of language (Labache et al., 2020; Villar-Rodríguez, Cano-Melle, et al., 2024; Villar-Rodríguez, Marin-Marin, et al., 2024). The corpus callosum plays a crucial role in the development of hemispheric specialization for language and is responsible for the transfer of information from one hemisphere to the other (Gazzaniga, 2000). Studies in children (Swanson, 2016) and adults (Paul et al., 2007) with agenesis of the corpus callosum have demonstrated its involvement in the development of language lateralization. Karolis et al. (2019) concluded that reduced lateralization of brain functions is associated with a larger corpus callosum volume. Building on this evidence, tasks requiring high processing speed and consequently greater interhemispheric connectivity may be linked to lower performance in individuals with atypical lateralization.

Our research proposal focuses on the potential association between the phenomenon of language lateralization and cognitive processing, as well as the exact role of the phenotype of atypical lateralization of language (bilateral or right language dominance) on the same cognitive tasks. Since the fMRI technique is crucial for identifying functional patterns (Johnstone et al., 2020), we used this method to categorize our sample into typical and atypical lateralization groups and into three groups (left language dominance, right language dominance and bilateral) based on their laterality index during a verb generation task. Then, we explored the performance in several cognitive functions, including reading, articulation, and verbal reasoning; visuospatial attention; working memory; and interference/inhibition. In line with previous studies, our hypothesis was that an atypical lateralization of language would be associated with a lower performance on language tests and tasks requiring rapid interhemispheric processing.

## MATERIALS AND METHODS

### Participants

Ninety-seven left-handed or mixed-handed individuals (53 with typical language lateralization and 44 with atypical language lateralization) who had previously participated in an fMRI session were invited to join a second behavioral session. The recruitment process for the first session consisted of an online screening in which the eligibility criteria were: (a) be over 16 years old; (b) write mainly with their left hand, as well as scoring above 32 on the Edinburgh Handedness Inventory (EHI; Oldfield, 1971) according to the scoring method by Bryden (1977); (c) have no history of head injury with loss of consciousness; (d) have no clinically relevant hearing or vision loss; (e) have no conditions incompatible with MRI imaging; and (f) have no diagnosed neurological or psychiatric disorders. Finally, a total of 86 healthy left-handers accepted the invitation. Participants ranged in age from 18 to 43 years old (*M* = 23.49, *SD* = 5.33) and in educational formation from 15 to 22 years (*M* = 18.81, *SD* = 1.86). For EHI and Laterality Quotient the score was: *M* = 42.63, *SD* = 4.99; and *M* = −73.89, *SD* = 25.76, respectively.

All methods were carried out in accordance with the guidelines and regulations approved by the Research Ethics Committee (CD/34/2019) of Jaume I University. Written informed consent was obtained from all participants following a protocol approved by the university. Before beginning the study, we provided all participants with a brief overview of the project and its objectives. Each participant received a financial compensation of 30 euros for their involvement.

### FMRI Procedure

We employed an fMRI-adapted verb generation task to classify participants based on their language lateralization. The index of lateralization was calculated using a formula explained below. This first session was conducted at the clinic ASCIRES-Castellón.

#### Image acquisition and processing

MRI scans were conducted using a 3T GE Signa-Architect scanner to collect structural and functional data. All slices were acquired through sagittal plane. For each participant, a 3D structural MRI was acquired through a T1-weighted magnetization gradient-echo sequence (TR/TE = 8.4/3.3 ms; flip angle = 12°; matrix = 512 × 512 × 384; voxel size = 0.47 × 0.47 × 0.5 mm). For the fMRI, a total of 144 functional volumes were recorded during the verb generation task using a gradient-echo T2-weighted echo-planar imaging sequence (TR/TE = 2,500/30 ms; flip angle = 70°; matrix = 64 × 64 × 30; voxel size = 3.75 × 3.75 × 4.5 mm; 144 strict axial slices).

#### Verb generation task and language lateralization analysis: Laterality index

The task consisted of a control condition, where participants read aloud letter pairs presented visually, and an activation condition where a noun was presented and participants had to say aloud the first verb that came to mind. Participants were instructed to respond by moving their jaws and vocalizing as little as possible to minimize head movements during scanning. To further reduce motion, each participant's head was carefully positioned within the coil and stabilized with foam pads. During practice trials, the experimenter verified that tasks were completed correctly. All stimuli were presented visually using MRI-compatible goggles, and each response was recorded with a noise-cancelling microphone. The paradigm used was identical to that of Villar-Rodríguez et al. (2020), a procedure that is very common in our research group.

The Statistical Parametric Mapping software package (SPM12; Ashburner et al., 2021) with default parameter settings was utilized for pre-processing and statistical analysis of the verb generation task. The steps followed were: (1) alignment of each fMRI sequence to the anterior commissure–posterior commissure (AC–PC) plane using the participant anatomical image; (2) correction for head motion, where functional images were realigned and resliced to align with the mean functional image; (3) co-registration with the mean functional image, transformation, and re-segmentation; (4) spatial normalization of the functional images to the MNI (Montreal Neurological Institute) space with a resolution of 3 mm^3^; and (5) spatial smoothing (full width half maximum = 4 mm).

We calculated the mean motion for each participant while inside the scanner and verified that there were no significant differences in movement between the groups, *F*(2, 82) = 2.97, *p* = 0.744, *η*_p_^2^ = 0.007. For this, we used the maximum and mean movement, based on the highest absolute values and the mean of the translation and rotation trajectories, using the absolute values of these parameters. Additionally, we individually checked that no subject had movement shifts greater than 2 mm.

For the first-level analysis, a general linear model was defined for the verb generation task. It was defined for each participant by contrasting the activation condition to the control condition. The blood oxygen level dependent signal was estimated by convolving the stimuli onset with the canonical hemodynamic response function. To account for motion-related effects, six motion-realigned parameters were included as covariates of no interest, obtained from head motion preprocessing. A high-pass filter (128 s) was applied to the functional data to remove low-frequency components. As a final step, we applied an inclusive mask to the results to include only the significant voxels that were most relevant in the verb generation task. This mask specifically targeted the inferior frontal region, including the pars opercularis and pars triangularis, defined based on standard anatomical criteria (Rutten et al., 2002). The mask was based on the Harvard-Oxford atlas (Rushmore et al., 2022).

Afterward, the resulting image was used to assess language lateralization for each participant through the bootstrap method implemented in the LI-toolbox, based on SPM (Wilke & Lidzba, 2007). The laterality index (LI) of language was calculated using the formula: [(L − R)/(L + R)] * 100; where L (left) and R (right) represent the number of significant voxels for each hemisphere. The LI represents the proportion of brain activity between the hemispheres during the verb generation task. LI values range from +100 (left language dominance) to −100 (right language dominance).

### Group Assignment

**Figure 1.**
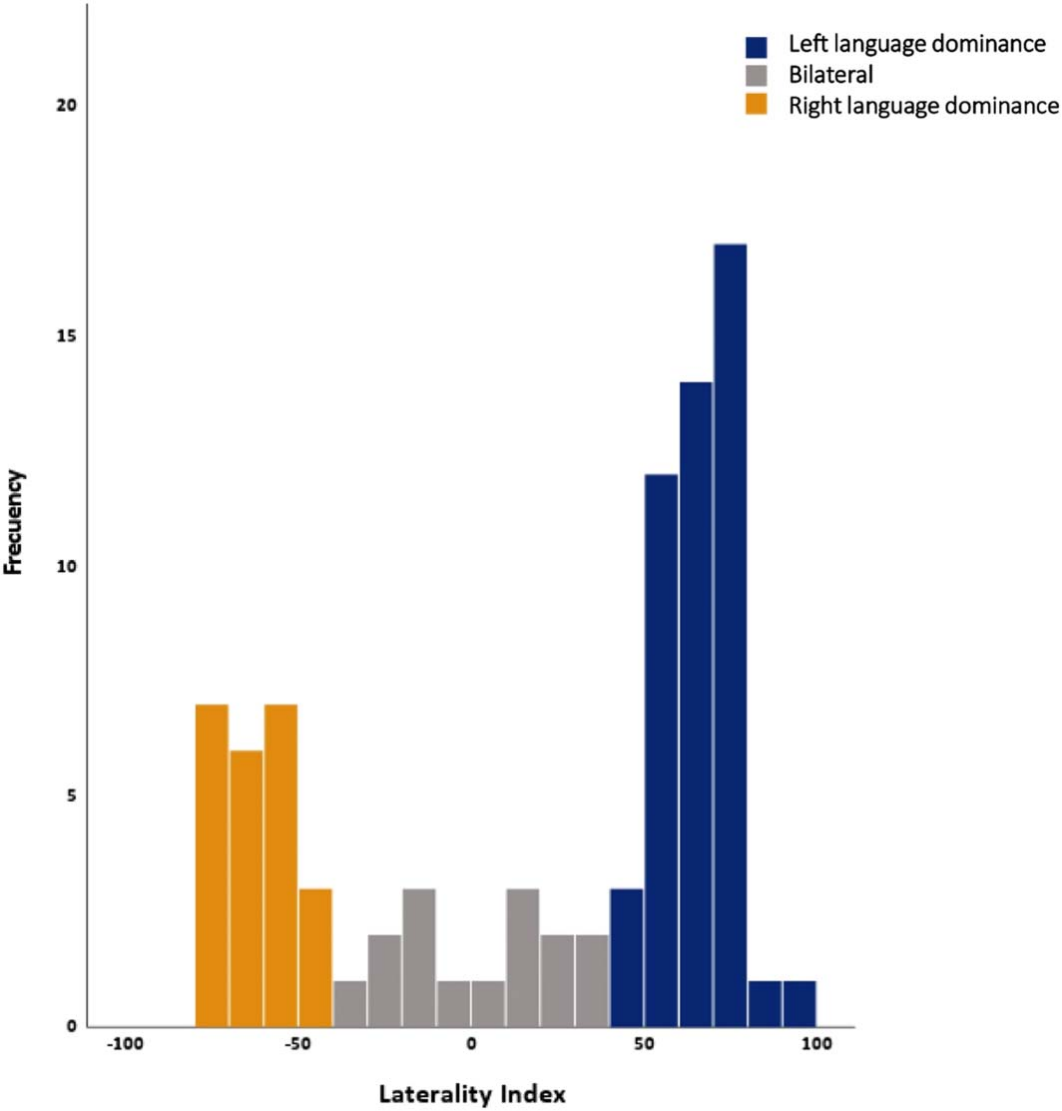
Distribution of laterality index derived from the verb generation task.

**Figure 2.**
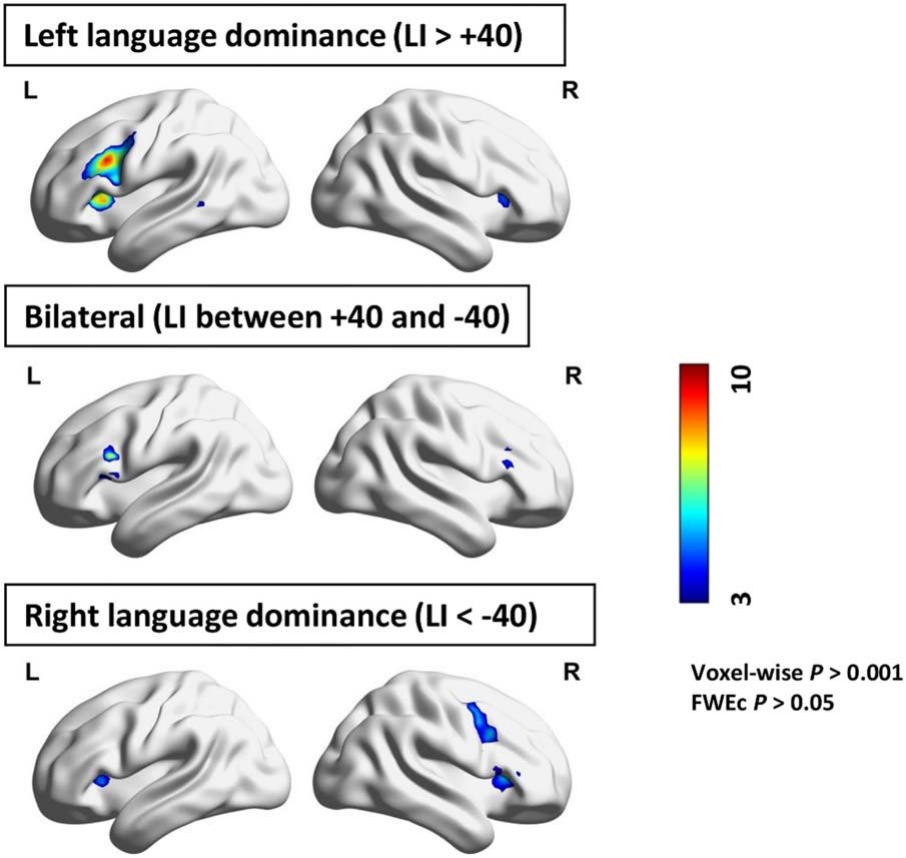
Group-wise activation during the verb generation task for the three lateralization groups. Color-bar represents *t* value. FWEc = Family-wise error corrected; LI = laterality index.

Based on previous studies (Labache et al., 2020; Mazoyer et al., 2014) that reported the importance of lateralization strength, and considering our objective to explore cognitive differences related to language lateralization, we determined that dividing participants into three language lateralization groups was the most appropriate approach. To achieve this, we opted for a more stringent cut-off point (+40/−40) compared to the traditionally used thresholds (+20/−20 or 0). This decision was based on previous studies mentioned which reported that a cut-off of 0.4—rather than 0.2—marks a significant change in the LI curve (Mazoyer et al., 2014). We believe this stricter threshold provides a clearer and more accurate definition of the lateralization groups. Participants were classified into two groups based on their LI in the verb generation task: typicals or left language dominance (LI ≥ +40) and atypicals (LI < +40). We also classified the sample in three groups (see Figure 1): left language dominance (LI > +40), right language dominance (LI < −40), and bilateral (LI between −40 and +40). Figure 2 shows the activation during the verb generation task for the three groups. There were no significant between-group differences in terms of age (*F*(2, 86) = 1.66, *p* = 0.19), sex (*χ*^2^ = 0.50; *p* = 0.77), or years of educational formation (*F*(2, 86) = 1.42, *p* = 0.246). However, significant differences were observed in the EHI scores (*F*(2, 86) = 3.83, *p* = 0.026), where both atypical lateralization groups (bilateral and right language dominance) obtained higher scores than the typical group. Also, the score of Laterality Quotient was significant (*F*(2, 86) = 4.2, *p* = 0.018), showing that the left language dominance group scored less in comparison with the other two lateralization groups. Table 1 presents all the descriptive statistics for the three lateralization groups.

**Table 1.** Means and standard deviations for the lateralization groups

	Left language dominance (*n* = 48)	Bilateral (*n* = 15)	Right language dominance (*n* = 23)
Sex (% females)	48.91	66.66	47.82
Age (yr)	22.9 ± 4.57	25.73 ± 6.64	23.6 ± 5.75
Edinburgh Handedness Inventory	41.37 ± 5.28	44.8 ± 4.18	43.83 ± 4.17
Laterality Quotient	−67.05 ± 28.66	−84.71 ± 17.83	−81.09 ± 19.29
Laterality index of language	64.58 ± 9.91	−2 ± 22.24	−62.92 ± 10.94
Years of educational formation	18.97 ± 1.98	19.13 ± 1.55	18.26 ± 1.76

### Cognitive Battery

This second session was conducted at Jaume I University (Castellón de la plana, Spain). We selected eight cognitive tasks that represent cognitive processes with dominance in the left hemisphere, right hemisphere, or no clear hemispheric dominance. We have emphasized tasks requiring faster processing speed. These tasks include: (1) reading a list of words and pseudowords (PROLEC; Cuetos et al., 2016) for reading; (2) a phoneme detection task for phonological processing; (3) a recall task of adding numbers (Paced Auditory Serial Addition Test [PASAT]; Tombaugh, 2006) for processing speed; (4) the *n*-back task for working memory; (5) a mental rotation task for exploring mental rotation and transformation; (6) a matrix reasoning test for visuospatial abstract reasoning, spatial perception, and the ability to mentally manipulate figures in space; (7) the stop-signal task for inhibitory control; and (8) the stroop task for interference during information processing.

#### Cognitive battery: Procedure

The behavioral session was held at Jaume I University, where participants completed the cognitive battery. E-Prime 2.0 software (Schneider et al., 2012) was used for the presentation of stimuli and behavioral data collection. Before beginning each task, the experimenter instructed participants about the rules they needed to follow during each task. Then, they completed a short practice session identical to the experimental task. For all tasks, the response keys used were “g” and “f.” Thus, participants were to press the key situated more to the right (“g”) to answer “yes” and the key closer to the left (“f”) to answer “no.” This response system was designed based on the manual preference of our sample, where all participants were left-handed, believing that this system would be a comfortable option for them.

#### Cognitive battery: Tasks

##### Reading: PROLEC battery.

Words and pseudowords lists were selected from the PROLEC battery to assess reading processes (Cuetos et al., 2016). These stimuli were presented and read in Spanish. Participants were instructed to orally read a total of six different lists, each containing 24 items: (1) familiar short words; (2) familiar long words; (3) unfamiliar short words; (4) unfamiliar long words; (5) short pseudowords; and (6) long pseudowords. Participants were required to read each list as quickly and accurately as possible. Once the participant finished reading a list, the experimenter presented the next list. Accuracy in reading was measured across the entire list, with incorrect or unintelligible responses not being accounted as correct. All lists were presented in the center of the screen, and a microphone was used to capture and record each response.

##### Phonological processing: Phoneme detection task.

We measured phonological processing via a phoneme detection task that required participants to promptly identify and verbally articulate the second phoneme of the presented word (Serrano & Defior, 2008). This word stimulus could be presented either in written format (displayed at the center of the screen) or in an auditory format (delivered through headphones). The task included 10 words (5 written and 5 auditory), featuring digraphs, words containing “h” (which is silent in Spanish), initial position consonant clusters, and syllabic structures of vowel-consonant-consonant. Similar to the reading task, immediately after the participant's response, the experimenter presented the next trial. Also, accuracy was registered and the incorrect responses or those unintelligible were not accounted as correct. All stimuli were presented in Spanish, and responses were recorded using a microphone.

##### Processing speed: PASAT test.

We measured processing speed via the PASAT. We used an auditory adaptation based on Tombaugh (2006). Participants listened to three lists of 18 numbers, with a number presented every 2 seconds. They were required to say aloud the result of the sum of the last two numbers they heard. For each trial, the experimenter annotated online whether the response was correct, incorrect, late, or an omission. Additionally, a microphone captured and recorded each response, and these recordings were later checked to verify the accuracy of the experimenter’s online annotations. An average of total hits was calculated considering the three lists.

##### Working memory: *N*-back test.

Working memory was assessed via a computerized *n*-back task based on Miró-Padilla et al. (2019), which included three conditions: two experimental conditions of working memory (2-back and 3-back) and one control condition (0-back). Stimuli consisted of different letters presented at the center of the screen every 500 ms. In the control condition, participants were required to press the “yes” button when the letter “X” appeared and the “no” button for other letters. In the experimental conditions, participants needed to press the “yes” button when the current letter shown on the screen matched the letter presented two or three trials back, and press “no” when the letters did not match. The task comprised a total of nine blocks of 30 trials, with three blocks for each level. The sequence of the stimuli was pseudo-randomized and was kept constant for all participants. Accuracy was registered across the three levels by calculating the percentage of correct responses.

##### Inhibitory control: Stop task.

Inhibitory control was measured via the stop-signal task. The task paradigm had two types of trials: Go and Stop. In the Go trials, participants were asked to respond by pressing the “g” or “f” key depending on whether a circle, triangle, square, or diamond appeared at the center of the screen. However, in 33% of the trials, a brief beep was played through the headphones just milliseconds before a geometric figure was presented, prompting participants that this was a Stop trial and hence they did not have to press any button. So, participants had to quickly and accurately discriminate the geometric figures but inhibit their response when the auditory signal was heard. They were asked to respond as quickly and accurately as possible and to avoid withholding their responses in anticipation of a possible beep.

The task consisted of four blocks, each containing 100 trials. An example of this paradigm can be found in Hall et al. (2022). In the stop-signal task, the stop-signal delay (SSD) dynamically adjusts after each trial based on the participant's performance. If the response is successfully inhibited, the SSD increases by 25 ms; if the inhibition fails, the SSD decreases by 25 ms. The SSD ranges between a minimum of 100 ms and a maximum of 800 ms. A shorter SSD makes it easier to inhibit the response, while a longer SSD makes it more challenging. This dynamic adjustment aims to standardize task difficulty across participants, targeting an optimal inhibition rate of around 50%. The objective was to obtain the stop-signal reaction time (SSRT; Verbruggen et al., 2019). This measure was calculated by subtracting the mean SSD in Stop trials from the median reaction times of correct Go trials.

##### Interference: Stroop test.

We administered three lists of the Spanish version of the Stroop test (Golden, 2020) to evaluate the processing of interferences. In the first list, the stimuli were color nouns, and participants were to read each word aloud. In the second list, “xxxx” was presented in various colors (green, red, yellow, or blue), and participants were required to say the color of each stimulus aloud. Finally, the last list was a combination of the first and second lists, where participants were instructed to say the color of the word instead of reading the word itself (which was a color noun). In all lists, participants were required to respond as quickly as possible, and each list had a time limit of 45 seconds. The experimenter registered the total of correct responses for each list. The results from the three lists were used to calculate an index of interference. The formulas used were as follow: (a) PC′ = C × P/C + P; and (b) PC − PC′ = Interference (P is list 1; C is list 2; PC is list 3; PC′ indicates average performance between list 1 and list 2). A higher number in the interference measure indicates greater cognitive interference).

##### Mental rotation: Mental rotation task.

Mental rotation and manipulation were assessed through a mental rotation task. The stimuli used were pairs of geometric figures (Shepard & Metzler, 1971) presented in 3D on the screen. The trials could be congruent (both figures were the same) or incongruent (both figures were different). Additionally, regardless of trial condition, the figures could appear in one of four types of spatial rotation (0°, 50°, 100°, 150°). Participants were instructed to respond as quickly as possible, although they were informed that they had time to mentally rotate, think, and decide their response. The maximum time allowed to respond was 7 seconds; if the participant did not respond within this time frame, the trial ended, and the next trial appeared immediately. There was a total of 96 trials, with half (48 trials) being congruent and the other half incongruent. The rotation variable was also controlled for each condition, with 12 trials for each combination of condition and rotation. The participants were required to press the “yes” button when figure pairs were the same and the “no” button when figure pairs were the different. We collected measures of accuracy for each condition.

##### Spatial reasoning: Matrix reasoning.

We measured spatial reasoning through the matrix reasoning subtest of the Weschler Preschool and Primary Scale of Intelligence—Fourth Edition (WAIS-IV; Wechsler, 2012). It consists of a series of visual problems in which individuals are presented with an incomplete matrix or a sequence of figures and must choose the piece that correctly completes the matrix or sequence from several available options. It is noteworthy that the difficulty increases progressively, and speed is not required, as participants have as much time as they need to respond to each trial. This task was administered manually by the researcher, who recorded the verbal responses of the participants.

### Statistical Analysis

All statistical analyses were conducted using IBM SPSS Statistics (Version 28). To identify the main cognitive factors and reduce the complexity of the data set for improved visualization and interpretation, we performed a principal component analysis (PCA). The Kaiser criterion (eigenvalue > 1) was applied to determine the number of factors to retain in the model. A Promax rotation was used, and the primary variables for each cognitive test were selected as follows: percentage of correct responses for word and pseudoword lists (reading), phoneme detection (phonological processing), PASAT (processing speed), mental rotation and 3-back (working memory), SSRT (ms) for the stop-signal task (inhibitory control), the WAIS-IV matrix reasoning subtest score (spatial reasoning), and the interference score for the Stroop test (interference control). We found that 3.77% of the behavioral data contained missing values. The causes included: 1.45% due to registration errors in software programs, 1.16% due to participant dropout during the evaluation, 0.87% due to negative SSRT scores (indicative of misunderstanding the instructions), and 0.29% due to color perception issues in one participant. To avoid excluding participants from the PCA, we replaced missing values with the mean scores for each task calculated from the rest of the sample. This imputation approach minimized data loss and ensured a more representative participant cohort.

Following factor analysis, we extracted the three factor scores corresponding to each dimension and conducted two multivariate analyses of covariance (MANCOVA). The first analysis compared typical and atypical lateralization groups, while the second compared the three lateralization groups (left language dominance, bilateral, and right language dominance). Given the broad age range of the sample, age was included as a covariate in both analyses. The data used has been deposited in OSF repository and is freely available for replication: https://osf.io/tvk4e/.

## RESULTS

### PCA Analysis

**Figure 3.**
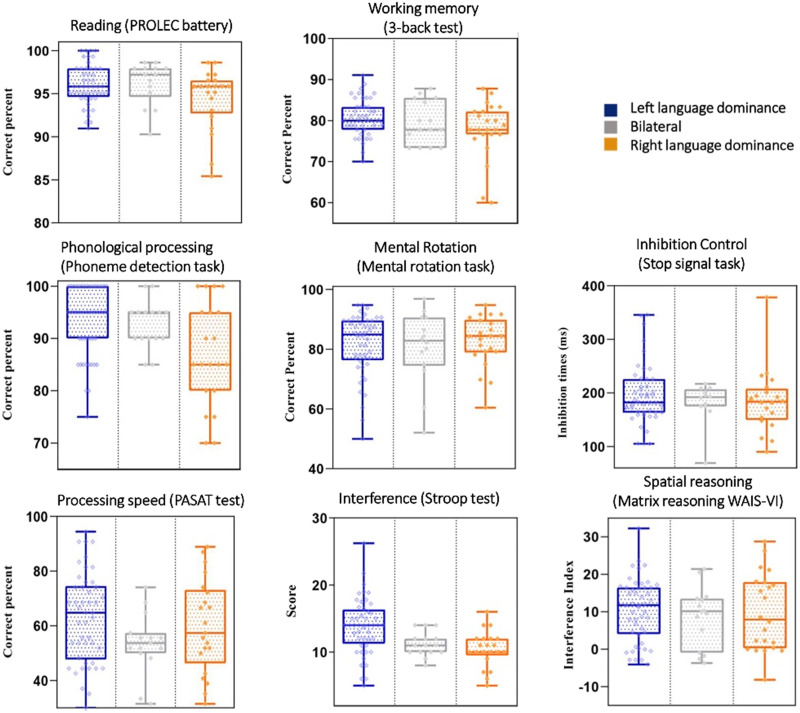
Distribution (boxplot) of cognitive tasks scores for each language lateralization group.

Means and standard deviations of cognitive tasks scores for each group can be found in Figure 3. A factor analysis was run including all these variables. The matrix model of PCA can be found in Table 2. Our PCA model had three principal factors that explained 56% of the total variance. The first component (28% of variance) was related to language and included high loadings for reading accuracy, second phoneme detection, and PASAT, which would represent skills in reading, articulation, and verbal reasoning. The second component (accounting for 15% of the variance) represented spatial abilities, as it included high loadings for mental rotation, matrix reasoning, and working memory. Lastly, the third component (13% of variance) represented the executive functions related to interference/inhibitory control, with high loadings for SSRT and Stroop interference. We identified an outlier in the third factor, defined as a score exceeding 3 standard deviations from the group mean. This data point was excluded from further analyses to prevent potential distortion of results.

**Table 2.** Matrix model with the three principal components

	Components		
	Language	Spatial	Executive control
Reading (accuracy % in prolec battery)	0.791	0.128	0.130
Phonological processing (accuracy % in phoneme detection task)	0.600	0.337	−0.149
Processing speed (accuracy % in PASAT test)	0.723	0.350	−0.056
Interference (scalar score in Stroop test)	0.477	0.065	−0.522
Inhibitory control (stop-signal task (ms))	0.048	−0.018	0.856
Mental rotation (accuracy % in mental rotation task)	0.102	0.780	−0.154
Spatial reasoning (scalar score in matrix reasoning WAIS-IV)	0.347	0.688	0.258
Working memory (accuracy % in *n* = 3 back test)	0.358	0.643	−0.043

*Note*. Values with loadings above 0.5 are shown in bold.

#### Typical vs. atypical

**Figure 4.**
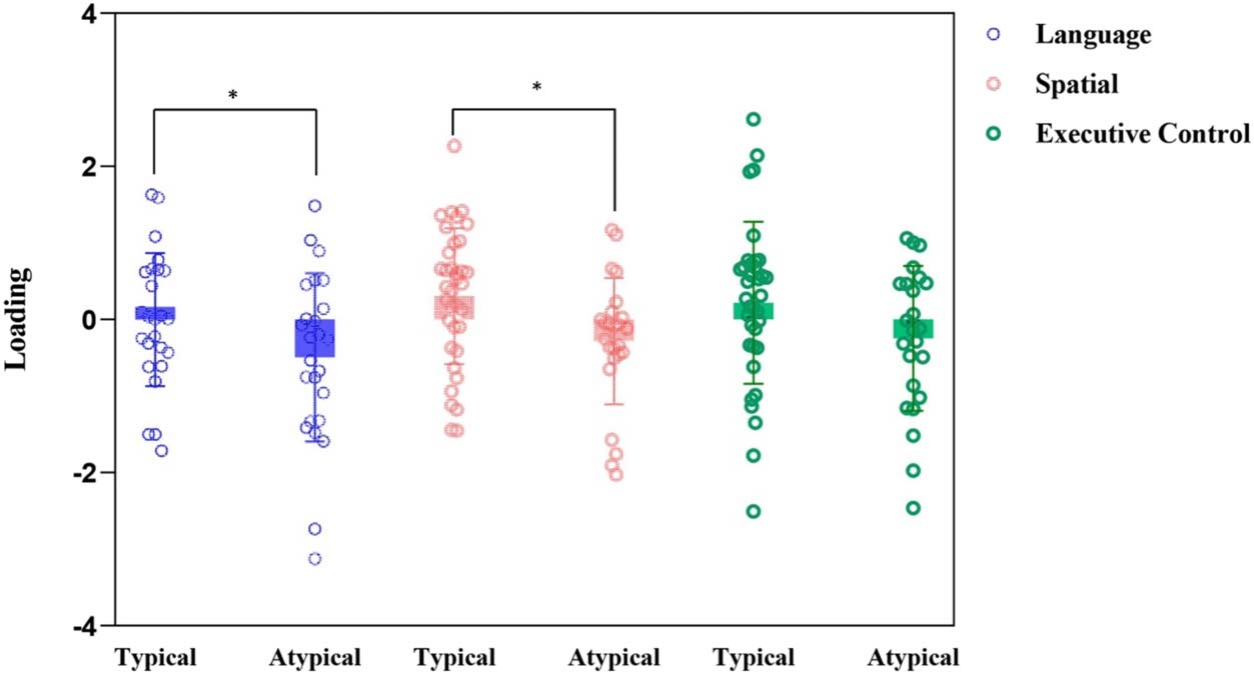
Boxplot of the score for each principal component across the three lateralization groups. Error bars represent the standard error of the mean. Asterisks represent statistically significant differences at *p* < 0.05.

The scores obtained for each factor were analyzed using a MANCOVA (see Figure 4). We found a main effect for the Atypical groups in the comparison (*F*(3, 79) = 4.32, *p* = 0.007, *η*_p_^2^ = 0.141), indicating that individuals with atypical lateralization performed worse than those with typical lateralization. Univariate analyses of the factor scores revealed significant effects of lateralization group for the Language factor (*F*(1, 84) = 6.02, *p* = 0.016, *η*_p_^2^ = 0.07) and the Spatial processing factor (*F*(1, 84) = 5.45, *p* = 0.022, *η*_p_^2^ = 0.06). The results for the third Executive factor only approached significance (*p* = 0.069).

#### Left language dominance vs. bilateral vs. right language dominance

**Figure 5.**
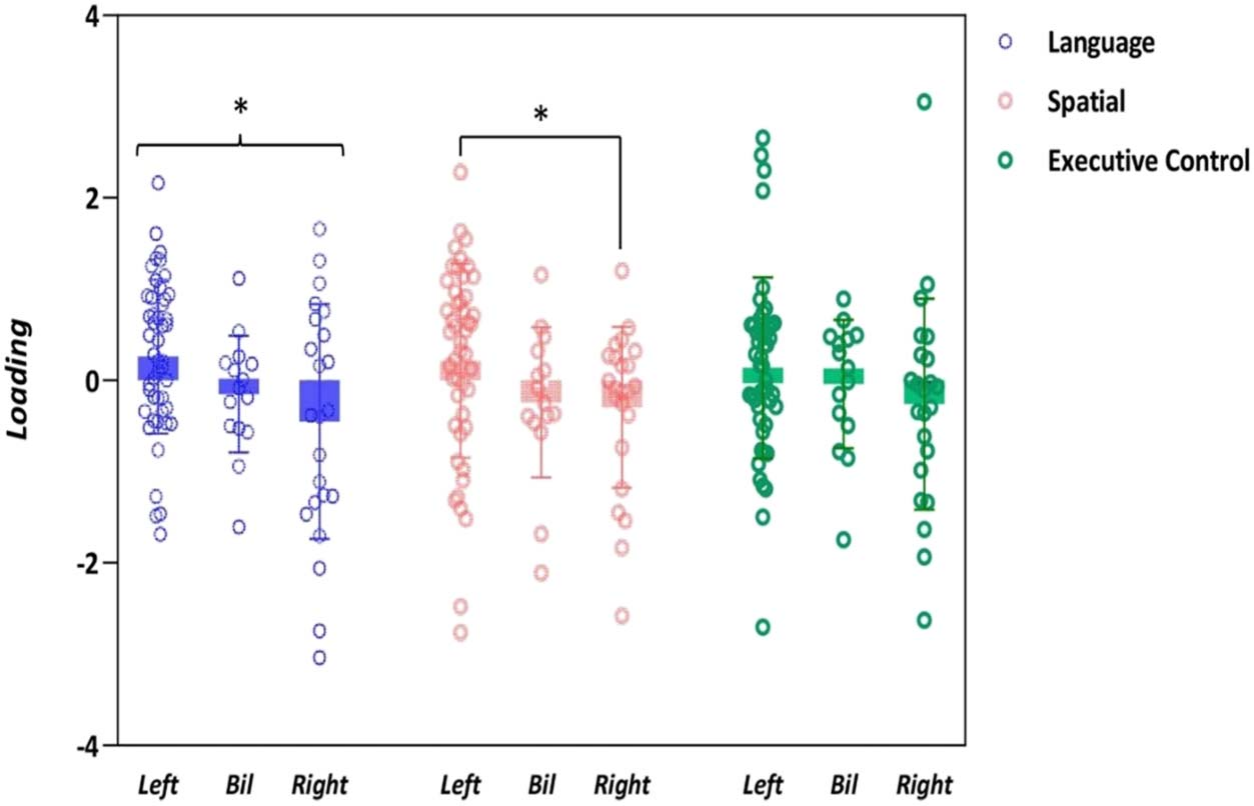
Boxplot of the score for each principal component across the three lateralization groups. Error bars represent the standard error of the mean. Asterisks represent statistically significant differences at *p* < 0.05.

The second MANCOVA analysis comparing the three lateralizations (see Figure 5), for which lateralization groups were used as a between-subjects factor, revealed a main effect for Group (Pillai’s Trace = 0.154, *F*(6, 158) = 2.20, *p* = 0.046, *η*_p_^2^ = 0.077). Post hoc analyses indicated that the left language dominance group outperformed both the right language dominance group (*p* = 0.001) and the bilateral group (*p* = 0.039). No significant differences were observed between the right language dominance and bilateral groups. Univariate analyses of the factor scores revealed a significant effect of lateralization group for the Language factor (*F*(2, 80) = 3.06, *p* = 0.06, *η*_p_^2^ = 0.071). The results for the Spatial processing factor approached significance (*F*(2, 80) = 2.71, *p* = 0.072, *η*_p_^2^ = 0.064).

## DISCUSSION

This study focuses on exploring how atypical lateralization of language in left-handers may lead to potential differences in cognitive performance. To investigate this, various tasks were used and classified into three different cognitive factors derived from PCA: reading, articulation, and verbal reasoning; spatial processing; and interference/inhibitory control. Our group and correlational results have shown that an atypical (especially right language dominance) lateralization of language was associated with lower scores on all cognitive tests including linguistic, spatial processing, and interference/inhibition tasks. These results highlight how a different cerebral organization (derived from an atypically lateralization of language) might be linked to slight cognitive differences.

**Figure 6.**
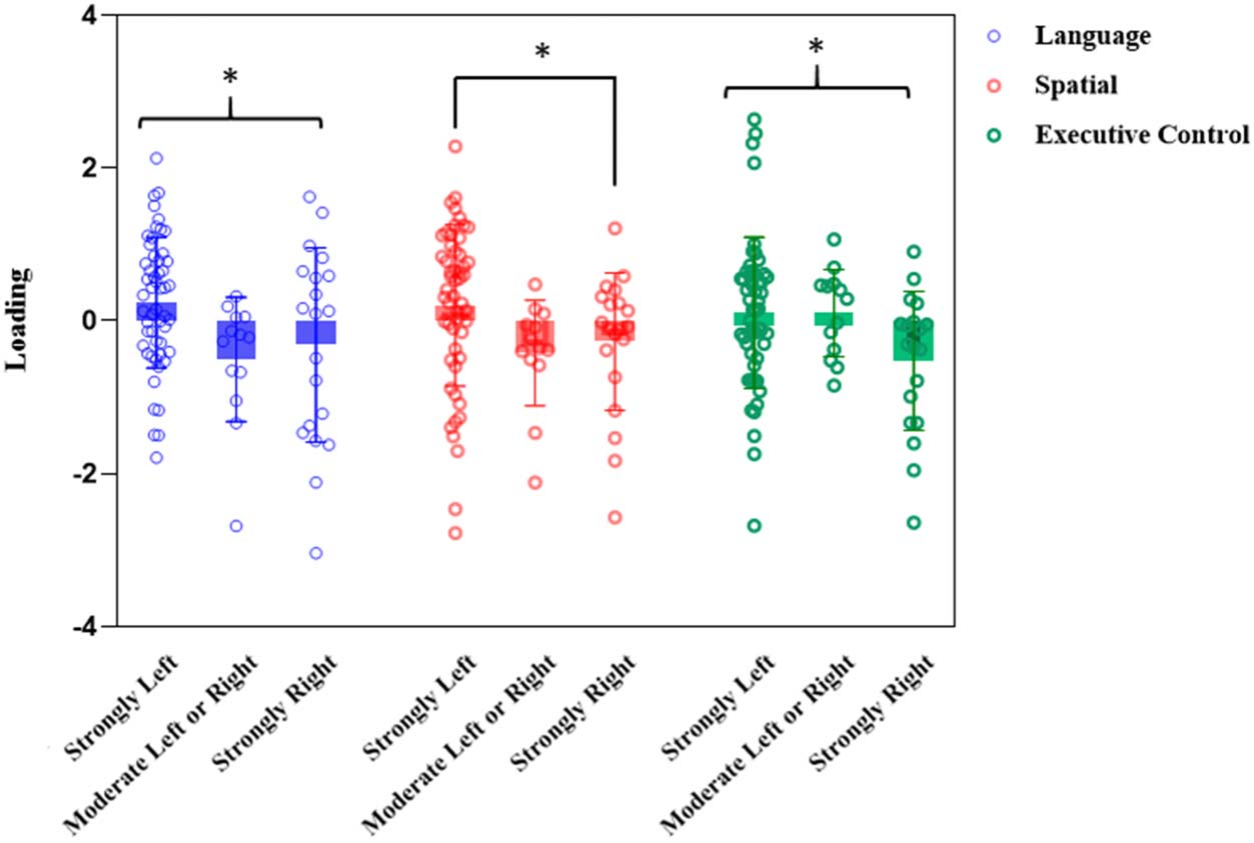
Boxplot of the score for each principal component across the three lateralization groups classified using cut-off scores employed by Mellet et al. (2014): left language dominance (LI > +19), right language dominance (LI < −51), and bilateral (LI between −50 and +18). MANCOVA analyses yielded significant effects for Language and Executive factors (*p* < −0.05). Error bars represent the standard error of the mean. Asterisks represent statistically significant differences at *p* < 0.05.

The results obtained in this study align with those that have demonstrated subtle cognitive difficulties in populations with atypical language lateralization (Mellet et al., 2014). The study that perhaps shows the most similar results to ours is that of Mellet et al. (2014), which reported poorer cognitive skills in both right-handers and left-handers with atypical language lateralization in three different factor scores related to memory, spatial processing, and language factors. However, they identified a small group of 10 individuals with a strong right hemisphere language lateralization who exhibited lower performance in the spatial factor but not in language (measured differently from our study; see below) or verbal memory (not assessed in our study). A simulation of our results using their cut-off points is presented in Figure 6, where the similarity to their findings can be observed. It is possible that the group with strong rightward lateralization has a mirrored brain organization (see Cai et al., 2013; Gerrits et al., 2019) that interferes less with cognitive task performance because they deviate less from a segregated distribution of cognitive functions, increasing the probability of crowding of the cognitive processes in the same hemisphere (Gerrits et al., 2020).

Let us analyze each factor separately. With regard to language skills in individuals with atypical language lateralization, previous studies have shown apparently disparate results, but perhaps not so much if we consider the tasks used to report them. In the study by Knecht et al. (2001), no relationship was found between language lateralization, naming, performance in word generation, and verbal IQ, in a sample of 326 adults (31 with weak lateralization and 31 with right lateralization). The finding of no relationship with verbal fluency has been confirmed in subsequent studies (Badcock et al., 2012; Flöel et al., 2001; Gerrits et al., 2020; Häberling et al., 2011; Lust, Geuze, Groothuis, & Bouma, 2011; Lust, Geuze, Groothuis, van der Zwan, et al., 2011). Thus, there appears to be no relationship between performance in fluency and other expressive tasks and language lateralization. Similarly, other studies have failed to find an association between atypical language lateralization and lower educational level (Flöel et al., 2005; Rosch et al., 2012; Whitehouse & Bishop, 2009). Therefore, previous studies do not show a relationship between language lateralization in adults and children in measures of verbal IQ or educational level.

On the other hand, atypical language lateralization appears to be a factor predisposing to lower reading and comprehension performance (Ocklenburg et al., 2024). In our study, the tasks with the highest load on the linguistic factor are word reading, second phoneme detection, and the PASAT test. These tasks are characterized by high temporal pressure, resulting in purely linguistic processes which are typically lateralized to the left hemisphere. Neuroimaging studies have shown that reading (Bonandrini et al., 2024), phonological awareness (Wang et al., 2021), and the PASAT test (Forn Frías, 2007) are cognitive processes that depend on both the left superior temporal gyrus and inferior frontal gyrus, as well as their connectivity. The observed association with atypical lateralization in the right hemisphere is consistent with Groen et al. (2012), where 58 children were studied, showing that those with atypical language lateralization had lower scores in vocabulary tasks and pseudoword reading (a trend was observed for word reading) compared to those with typical lateralization. Based on previous literature, our results provide the first evidence with this pattern in a sample of healthy left-handed adults with right-hemisphere language lateralization. Our findings reflect a negative trend in cognitive performance in language skills in right language dominance individuals compared to those with left language dominance lateralization, particularly in tasks predominantly focused on reading, articulation, and verbal reasoning.

Our findings are also in line with results in children, associating right structural asymmetries in frontal language areas with lower language performance (Eichner et al., 2024; Qi et al., 2019). Therefore, the language limitations in left-handed adults do not seem to be associated with weak language lateralization (Bishop, 2013), but rather with structural asymmetry that results in less left-hemisphere involvement in controlling the function. The left hemisphere is typically the dominant one for language in the majority of individuals (Mazoyer et al., 2014; Pujol et al., 1999; Springer et al., 1999; Szaflarski et al., 2002). The reduced involvement of this hemisphere may be the result of the lack of a typical neurodevelopment, which leads to a shift from interhemispheric to intrahemispheric processing (Friederici et al., 2011). In fact, recent studies have described neural mechanisms potentially behind atypical language lateralization, which could be interpreted as a reduced shift from interhemispheric to intrahemispheric processing, either due to underdevelopment of the arcuate fasciculus or overdevelopment of the corpus callosum (Labache et al., 2020; Villar-Rodríguez, Marin-Marin, et al., 2024; Zhu et al., 2025). This anomalous development of the structures supporting language lateralization might be responsible for the cognitive differences between typical and atypical left-handers, maybe through more bilateral language networks in some or all of its cognitive components (Bradshaw et al., 2020).

A second finding of this study is that the right language dominance group shows worse performance in visuospatial processing tasks, including *n*-back, compared to the left language dominance group. Previous studies exploring visuospatial performance have used the line bisection task, not finding any association between lateralization and performance (Cai et al., 2013; Filardi, 2019; reviewed by Quin-Conroy et al., 2024). This discrepancy with our results might stem from the different characteristics of the tasks employed. The line bisection task is strongly lateralized in the right hemisphere (Seydell-Greenwald et al., 2019). However, our three visuospatial tasks (the ones with the highest loadings) share a common bilateral parietal control, as demonstrated by several neuroimaging studies for mental rotation (for a review, see Hiew et al., 2023), *n*-back (Owen et al., 2005), and the matrix reasoning test (Caudle et al., 2023). Therefore, even though these processes have a clear bias toward the right hemisphere (because of their visuospatial component) they also require an important bilateral processing where corpus callosum plays an important role, potentially explaining the worse execution among an atypically right-lateralized population. In this line, the results by Mellet et al. (2014) also showed a worse execution (although nonsignificant) among the rightward individuals—using similar tests to ours, such as mental rotation or Raven’s matrices, together with other tests such as the Corsi block-tapping task or the maze test. Therefore, an atypical control of language does not seem to be beneficial to the processing of some visuospatial tasks requiring collaboration between both hemispheres (Labache et al., 2020; Villar-Rodríguez, Marin-Marin, et al., 2024).

Lastly, the lateralization groups did not differ significantly in the executive component, which includes tests of inhibitory control and cognitive interference. These tasks require active participation of the frontal lobe bilaterally (Aziz-Safaie et al., 2024), but this factor did not seem to be influenced by different frontal language lateralization. In the case of the stop-signal task, the results are consistent with our previous study, in which we showed that atypical lateralization of both language and inhibitory control did not affect task performance (Villar-Rodríguez, Cano-Melle, et al., 2024). Despite the absence of differences in the univariate analyses, considering the overall results and especially Figure 6 based on the classification by Mellet et al. (2014), it can be speculated that strong rightward lateralization (but not bilaterality) predisposes individuals to poorer performance on these tests.

Although our study presents a higher number of subjects with atypical language laterality (right and bilateral) compared to previous studies, it is still far from being a representative cohort. Hence, given the high performance of our participants, and the low observed effects, our results should be interpreted with caution. Therefore, it would be interesting to conduct this study with an even larger sample of participants with atypical language lateralization, as well as consider increasing the cognitive demands of the tasks. The lack of consistency in the measurement of laterality in the literature could explain the limited robustness of the results so far, as well as some discrepancies between previous findings and certain results reported in this manuscript. In our study, language laterality was determined using a verb generation task, which functionally involves anterior brain regions. A valuable extension of this work would be to replicate the study, assessing the laterality of other aspects of language, such as comprehension, which may involve lateralization networks different from those engaged in language production (COLA consortium et al., 2022; Vingerhoets, 2019). It would also be interesting to replicate this study with a focus on the brain organization of two or more components of language and explore the cognitive impact of the hemispheric convergence or divergence between these components. Based on the new theoretical frameworks (Gerrits, 2024; McManus, 2022), it would also be interesting to explore lateralization patterns of other cognitive functions and compare them with cognitive performance (Villar-Rodríguez, Davydova, et al., 2024).

In summary, our study hints at cerebral lateralization of language having significant effects on the performance of different cognitive tasks. The novel findings indicate that left-handed adults with right language lateralization exhibit a diminished performance overall but especially in certain reading, articulation and verbal reasoning, and spatial tasks. However, the effect of atypical language lateralization on executive tasks needs to be further investigated in the future. In line with recent studies (Ocklenburg et al., 2024), our results further support the idea that atypical lateralization could serve as a biomarker indicative of modifications in cognitive processing, but it should not necessarily be considered a definitive diagnosis, as atypical lateralization does not always lead to a global negative impact or large cognitive differences.

## ACKNOWLEDGMENTS

We express our gratitude to all the participants for their collaboration in this study, as well as the radiographers at the clinic ASCIRES-Castellón for their valuable assistance during data acquisition.

## FUNDING INFORMATION

Cristina Cano-Melle, Ministerio de Ciencia, Innovación y Universidades (https://dx.doi.org/10.13039/100014440), Award ID: PRE2020-096420. César Avila, Universitat Jaume I (https://dx.doi.org/10.13039/501100004834), Award ID: UJI-B2021-11. César Avila, Agencia Estatal de Investigación (https://dx.doi.org/10.13039/501100011033), Award ID: PID2019-108198GB-I00. César Avila, Agencia Estatal de Investigación (https://dx.doi.org/10.13039/501100011033), Award ID: PID2023-150776NB-I00. César Avila, Prometeo program of the Generalitat Valenciana, Award ID: CIPROM/2023/58.

## AUTHOR CONTRIBUTIONS

**Cristina Cano-Melle**: Data curation: Equal; Formal analysis: Equal; Investigation: Equal; Methodology: Equal; Software: Equal; Writing – original draft: Equal; Writing – review & editing: Equal. **Esteban Villar-Rodríguez**: Data curation: Supporting; Investigation: Supporting. **María Baena-Pérez**: Data curation: Supporting; Investigation: Supporting. **María Antonia Parcet**: Conceptualization: Supporting; Writing – review & editing: Supporting. **César Avila**: Conceptualization: Lead; Methodology: Lead; Project administration: Lead; Supervision: Lead; Writing – original draft: Lead; Writing – review & editing: Lead.

## DATA AVAILABILITY STATEMENT

The data used in this study has been deposited in OSF repository and is freely available for replication: https://osf.io/tvk4e/.

## TECHNICAL TERMS

Hemispheric lateralization: Functional specialization of one brain hemisphere (left or right) for specific tasks like language, motor control, or spatial perception.
Left-handedness: Natural preference for using the left hand for tasks such as writing and eating, linked to differences in brain organization.
Visuospatial: Related to perception and processing of visual and spatial information, such as orientation, object location, and spatial relationships.
Functional magnetic resonance imaging (fMRI): Neuroimaging technique measuring blood flow changes in the brain, reflecting neuronal activity during cognitive tasks or stimuli.
Phenotypes of atypical language: Observable variations in brain organization and language performance deviating from typical development due to genetic or neurological factors.
Verb generation task: Neuropsychological test where participants generate a verb associated with a given noun, activating brain regions involved in language (e.g., Broca’s area).
Edinburgh Handedness Inventory (EHI): Standardized questionnaire measuring hand preference (left, right, or ambidextrous) across various activities, producing a quantitative laterality index.
Executive control: Set of cognitive processes regulating thoughts and actions, including planning, inhibition, decision-making, and impulse control.
Cognitive skills: Mental abilities required for thinking, learning, problem-solving, and processing information such areas as memory, attention, and reasoning.
